# Long non-coding RNA TTTY15 silencing inhibits gastric cancer progression by sponging microRNA-98-5p to down-regulate cyclin D2 expression

**DOI:** 10.1080/21655979.2022.2047398

**Published:** 2022-03-10

**Authors:** Xigang Wen, Wenling Han, Chao Liu

**Affiliations:** a Department of Gastrointestinal Surgery, The Third People’s Hospital of Hubei Province, Wuhan, China; b Department of Hospital Infection Office, The Third People’s Hospital of Hubei Province, Wuhan, China

**Keywords:** LncRNA *TTTY15*, *miR-98-5p*, *CCND2*, gastric cancer

## Abstract

Gastric cancer is the most common malignant tumor in the digestive system. However, the detection rate of early gastric cancer is low, resulting in delayed prognosis and poor outcomes. The identification of effective therapeutic targets for gastric cancer is, therefore, of profound significance. Recently, various lncRNAs have been shown to be biomarkers for different cancers. This study investigated the role of long non-coding RNA (lncRNA) *TTTY15* in gastric cancer. The expression level of *TTTY15, miR-98-5p*, and *cyclin D2* (*CCND2*) were evaluated by quantitative reverse transcription polymerase chain reaction (qRT-PCR) and Western blot assay using tumor and non-tumor tissues collected from 30 patients with gastric cancer, gastric cancer cell lines (AGS, SNU-5, and NCI-N87), and the normal gastric epithelial cell line GES-1. The interaction between *TTTY15* and *miR-98-5p* and between *miR-98-5p* and *CCND2* were predicted by bioinformatics and then further verified by dual-luciferase and RNA pull-down analyses. Cell proliferation was evaluated by 3-(4,5-dimethylthiazol-2-yl)-2,5-diphenyl-2 H-tetrazolium bromide (MTT) assay, and apoptosis was measured using flow cytometry and caspase-3 assay. The results indicate that *TTTY15* and *CCND2* expression increased and *miR-98-5p* expression decreased in gastric cancer tumor tissues and cell lines. *TTTY15* knockdown inhibited gastric cancer cell proliferation but promoted apoptosis by sponging *miR-98-5p*, which acted as a tumor suppressor gene by reducing the expression of its target gene *CCND2* in gastric cancer. In conclusion, lncRNA *TTTY15* is a potential oncogene involved in gastric cancer and may be a novel therapeutic target for gastric cancer treatment.

## GRAPHICAL ABSTRACT

**Figure:**
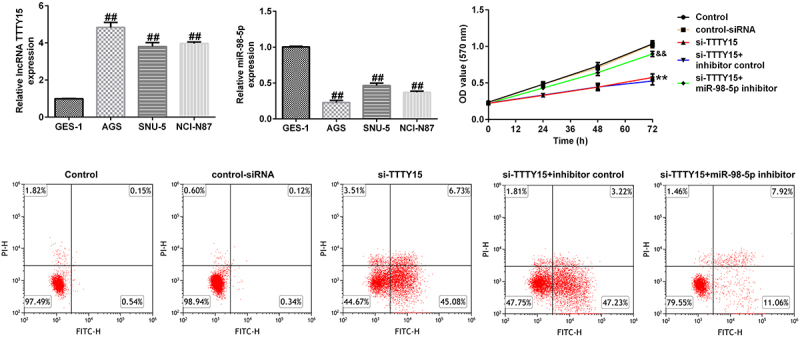

## Introduction

Gastric cancer, a common type of malignancy, currently, has the third-highest incidence and fifth-highest mortality rate in the world [1,2]. In China, gastric cancer has become the second most common type of malignant tumor after lung cancer. An increasing number of studies have confirmed that gastric cancer is often caused by *H. pylori* infection, smoking, consumption of cured foods, and a high-salt diet [3]. At present, surgery combined with radiotherapy, immunotherapy, and targeted therapy is still the most effective way to treat gastric cancer. However, due to atypical early symptoms and the relatively limited awareness of the importance of medical checkups, the detection rate of early gastric cancer is significantly lower in China than in Europe and America [4]. The identification of effective therapeutic targets of gastric cancer is, therefore, of profound importance for its diagnosis and treatment.

Approximately 98% of the human genome is thought to not encode proteins and is hence classified as non-coding genes [5]. Long non-coding RNAs (lncRNAs) are a class of non-coding genes that are more than 200 nucleotides in length [6]. Accumulating evidence has shown that lncRNAs can modulate tumor formation and development in various cancers [7,8]. Moreover, the role and mechanism of lncRNAs in gastric cancer has become a prominent topic in academic research [9,10]. *TTTY15* has been reported to be overexpressed and to promote tumor formation in esophageal squamous cell carcinoma [11], colorectal cancer [12], prostate cancer [13], and lung cancer [14]. However, how *TTTY15* is involved in gastric cancer remains unclear.

MicroRNAs, a type of small non-coding RNAs, are implicated in regulation of cell differentiation, metabolism, and the cell cycle by reducing target gene expressions at the post-transcriptional level [15,16]. The study of miRNAs in oncology started with chronic granulocytic leukemia (CML), with *miR-15a* and *miR-16-1* being reported to be down-regulated in CML [17,18]. Subsequently, scientists have analyzed and compared miRNA expression profiles in breast cancer [19], lung cancer [20], prostate cancer [21], ovarian cancer [22], and gastric cancer [23]. Thus, miRNAs appear to play crucial regulatory roles in tumorigenesis and development. This study aimed to investigate the mechanism of *miR-98-5p* involvement in gastric cancer.

In this study, we hypothesized that *TTTY15* plays a role in gastric cancer by regulating *miR-98-5p/CCND2*. Therefore, this study was designed to explore the role and molecular mechanisms of *TTTY15* in gastric cancer cells.

## Materials and Methods

### Patient enrollment

A total of 30 paired tumor tissues and adjacent normal tissues that were more than 2 cm from the tumors [24] were collected from patients who underwent surgical treatment for gastric cancer at the Third People’s Hospital of Hubei Province between January 2019 and March 2021. All excised specimens were stored at −80°C until further use. None of these patients had undergone adjuvant treatment such as chemotherapy or radiotherapy before surgery. All the patients signed the informed consent documents. The present study was approved by the Ethical Review Committee of the Third People’s Hospital of Hubei Province.

### Cell culture

AGS, SNU-5, and NCI-N87 gastric cancer cell lines and the gastric epithelial cell line GES-1 were purchased from the Chinese Academy of Sciences (Shanghai, China). All cells were cultured in Roswell Park Memorial Institute (RPMI)-1640 medium containing 10% fetal bovine serum (FBS) in a humidified incubator at 37°C in the presence of 5% CO_2_.

### Cell transfection

2 μM lncRNA TTTY15-siRNA (si-TTTY15), 2 μM control-siRNA, 50 nM miR-98-5p inhibitor, 100 nM miR-98-5p mimic, 50 nM inhibitor control, and 100 nM mimic control were synthesized using RiboBio Co. (Guangzhou, China). The adenovirus plasmid for overexpression of CCND2 (10 μl) and the negative control plasmid (10 μl) were obtained from Santa Cruz Biotechnology (USA). These oligonucleotides were transfected into AGS gastric cancer cells using Lipofectamine 3000 reagent (Thermo Fisher Scientific, Inc., Shanghai, China) according to the manufacturer’s instructions.

### Binding site prediction

The online tool starBase was used to predict the potential targets of TTTY15 and the binding sites between TTTY15 and miR-98-5p [25]. Similarly, the bioinformatics tool TargetScan (http://www.targetscan.org/) was used to predict the downstream targets of miR-98-5p and their binding sites [26].

### Dual-luciferase reporter assay

The TTTY15 sequences containing the potential miR-98-5p binding sites was amplified by PCR and subcloned into a pGL3 vector (Promega, USA), designated as WT-TTTY15 plasmid. Additionally, MUT-TTTY15, which did not contain the bindings sites, was constructed by GenePharma (Shanghai, China). AGS cells were seeded in 24-well plates and transfected with WT-TTTY15 or MUT-TTTY15 plasmids and miR-98-5p mimic or mimic control using Lipofectamine 2000 (Thermo Fisher Scientific, Inc., Shanghai, China) according to the manufacturer’s instructions. After 24 h of cultivation at 37°C with 5% CO2, the transfected cells were harvested and the luciferase signal was measured using a Promega Kit (Promega, USA) with a GloMax® 2020 Single Tube Luminometer instrument (Promega, USA). The ratio of firefly luciferase activity was normalized to Renilla luciferase [27].

### RNA pull-down assay

AGS cells were transfected with lncRNA TTTTY15 biotinylated at the 3′ end, or Oligo probe, and cultured at 37°C with 5% CO_2_. The transfected cells were then cultured in the presence of streptavidin-coated magnetic beads (Gzscbio, Guangzhou, China). Finally, the biotin-coupled RNA complex was used to determine the abundance of miR-98-5p by qRT-PCR analysis of the bound fractions [28].

### Quantitative reverse transcription polymerase chain reaction (qRT-PCR) analysis

Total RNA was extracted from all the samples using Trizol reagent. After measuring the RNA concentration using Nanodrop 2000, 2 μg RNA was reverse transcribed into cDNA using a kit from Takara Co. (RR003A; Japan). The cDNA obtained by reverse transcription was subjected to real-time fluorescence quantification of gene expression on StepOnePlus™ Real-Time PCR System (Applied Biosystems; Shanghai, China) using a Realtime PCR Master Mix kit (QPK-201; Toyobo, Japan). The PCR thermal cycles comprised initial denaturation at 95°C for 5 min; followed by 40 cycles of 15 sec at 95°C, 1 min at 60°C and 30 sec at 72°C; and a final extension for 10 min at 72°C. At the end of the PCR reaction. CT values were derived from the PCR software for relative quantification using the 2^−ΔΔCT^ method [29]. GAPDH/U6 was used as an internal reference. Primer sequences were listed as following:

TTTY15, forward 5′-TGAGGGAGGGAT GTAGCTTT-3′;

reverse 5′-GAAGTCAAGCAGGCAACTGA-3′;

miR-98-5p, forward 5′-TGAGGTAGTAGTTTGTGCTGTT-3′;

reverse 5′-GCGAGCACAGAATTAATACGAC-3′;

GAPDH, forward 5′-CATCATCCCTGCCTCTACTGG-3′;

reverse 5′-GTGGGTGTCGCTGTTGAAGTC-3′;

U6 S 5′-GGAACGATACAGAGAAGATTAGC-3′;

Stem-loop-R 5′-CTCAACTGGTGTCGTGGAGTC-3′;

PCNA, forward 5′- CCTGCTGGGATATTAGCTCCA-3′;

reverse 5′-CAGCGGTAGGTGTCGAAGC-3′;

CCND2, forward 5′-ACCTTCCGCAGTGCTCCTA-3′;

reverse 5′-CCCAGCCAAGAAACGGTCC-3′.

### MTT assay

MTT assay was used to evaluate cell proliferation. Briefly, transfected AGS cells were seeded at 1 × 10^4^ cells/well in 96-well plates and cultured at room temperature in a humid atmosphere with 5% CO_2_. At different time points (24, 48, and 72 h), 20 μL of MTT solution (Procell, Wuhan, China) was added to each well and the plates were incubated for 2 h at 37°C with 5% CO_2_. Then, 150 μL of dimethyl sulfoxide (Procell, Wuhan, China) was added to dissolve the formazan. Finally, the absorbance was measured at 570 nm using a microplate reader [30].

### Flow cytometry analysis

After transfection, 1 × 10^5^ AGS cells/well were cultured in 96-well plates at 37°C with 5% CO_2_ for 24 h. Then, 5 μL of annexin-V FITC and 10 μL of propidium iodide staining solution (PI) were added to each well and incubated for 15 min at room temperature in the dark. The labeled cells were then placed on ice and subjected to flow cytometry using a BD FACSCalibur (San Diego, CA, USA) [31]. The number of apoptotic cells was determined using ImageJ software.

### Caspase-3 activity

After transfection, the AGS cells were seeded into 6-well plates and cultured at 37°C in the presence of 5% CO_2_. Then, the cells were harvested by centrifugation to collect the supernatant fluid. The caspase-3 activity in the supernatant fluid was assayed with a microplate reader at 405 nm using a caspase-3 activity detection kit (Beyotime Institute of Biotechnology, Shanghai, China) according to the supplier’s instructions [32].

### Western blot assay

Protein expressions were determined using Western blot assay [33]. Total protein was extracted using RIPA cell lysate buffer containing a protease inhibitor cocktail. The protein concentration was then measured using the BCA method. The proteins were separated by SDS-PAGE gel electrophoresis and transferred to PVDF membranes. The membranes were then blocked with 5% BSA at room temperature for 50 min to eliminate nonspecific binding of primary and secondary antibodies. Primary antibody (cleaved-Caspase3 antibody, ab32042, 1:1000, Abcam; GAPDH antibody, ab9485, 1:1000, Abcam; PCNA antibody, ab18197, 1:1000, Abcam; CCND2 antibody, ab207604, 1:1000, Abcam;) was then added to the PVDF membrane and incubated overnight at 4°C. The next day, the membranes were probed with the corresponding HRP-labeled secondary antibody (ab7090, 1:1000, Abcam) by incubation at room temperature for 2 h. ECL chromogenic solution A and B were mixed, added dropwise to the position of the target band on the membrane, and photographed using ImageJ software.

### Data analysis

All statistical analyses were performed using SPSS 20 statistical software (SPSS, Chicago, IL, USA) and GraphPad Prism 5 (GraphPad Software, La Jolla, CA, USA). Data are presented as means ± SD. Student’s *t*-test or one-way ANOVA analysis followed by Tukey’s test were used for the statistical analysis. A p-value of less than 0.05 (*P* < 0.05) was considered statistically significant.

## Results

### lncRNA TTTY15 sponged miR-98-5p in AGS cells

**Figure 1.**
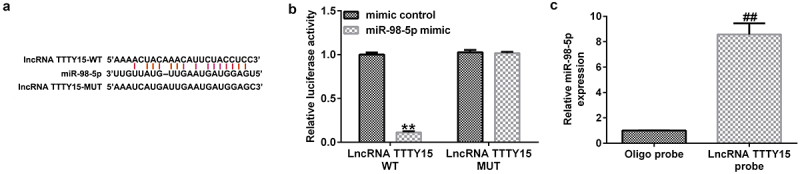
lncRNA TTTY15 sponges miR-98-5p in gastric cancer.

First, the starBase online tool was utilized to predict the potential targets of lncRNA TTTY15. This tool identified the potential binding sites between the 3′-UTR of TTTY15 and miR-98-5p (Figure 1a). Furthermore, using a dual-luciferase reporter assay, it was found that the luciferase activity was greatly decreased in the miR-98-5p mimic and WT-TTTY15 co-transfection groups compared with that in mimic control group (Figure 1b). Additionally, the RNA pull-down assay also demonstrated that the expression of miR-98-5p was greatly enhanced in AGS cells treated with lncRNA TTTTY15 probes compared with those treated with NC probes, (Figure 1c). These results suggest that lncRNA TTTY15 can sponge miR-98-5p in AGS cells.

(A) starBase predicted the binding sites between miR-98-5p and lncRNA TTTY5. (B) Dual-luciferase reporter assay verified the target relationship between miR-98-5p and lncRNA TTTY5. (C) RNA pull-down analysis revealed the targeted relationship between miR-98-5p and lncRNA TTTY5.

### *Enhanced lncRNA* TTTY15 *and decreased* miR-98-5p *expression in gastric cancer patients and cell lines*

**Figure 2.**
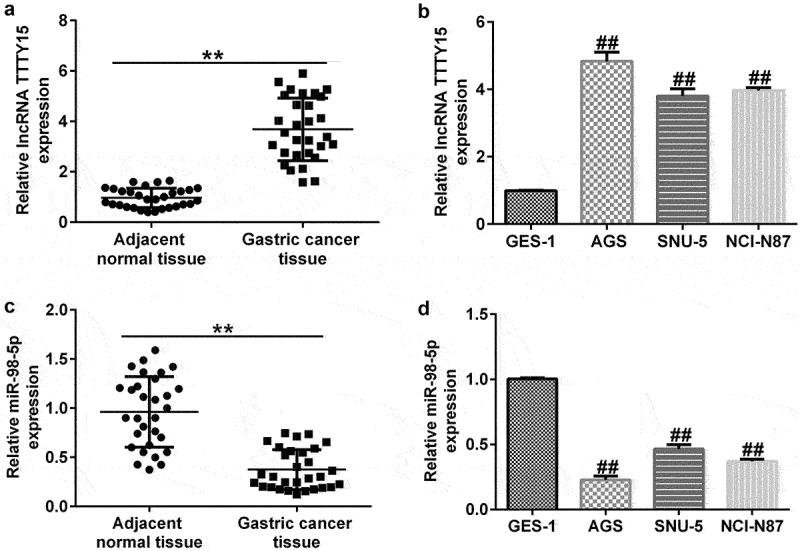
Ectopic expressions of lncRNA TTTY5 and miR-98-5p in gastric cancer tissues and cells.

To further investigate the mechanisms underlying lncRNA TTTY15 and miR-98-5p involvement in gastric cancer, we used qRT-PCR analysis to distinguish aberrantly expressed lncRNA TTTY15 and miR-98-5p. lncRNA TTTY15 expression was greatly increased and miR-98-5p expression was down-regulated in tumor tissues compared with that in non-tumor adjacent tissues (Figure 2a and 2C). Similarly, lncRNA TTTY15 levels were increased and miR-98-5p levels were reduced in the gastric cancer cell lines AGS, SNU-5, and NCI-N87 compared with that in the normal gastric epithelial cell line GES-1, (Figure 2b and 2D). The changes in lncRNA TTTY15 and miR-98-5p levels were most obvious in AGS cells, which were hence chosen for the subsequent experiments.

(A) lncRNA TTTY5 levels in tumor tissues and non-tumor tissues obtained from 30 patients with gastric cancer. (B) Aberrant expressions of lncRNA TTTY5 in the GES-1 cell line and gastric cancer cell lines (AGS, SNU-5, and NCI-N87). (C) miR-98-5p levels in tumor tissues and non-tumor tissues obtained from 30 patients with gastric cancer. (D) Aberrant expressions of miR-98-5p in the GES-1 cell line and gastric cancer cell lines (AGS, SNU-5, and NCI-N87).

### lncRNA TTTY15 silencing inhibited AGS cell proliferation by sponging miR-98-5p

**Figure 3.**
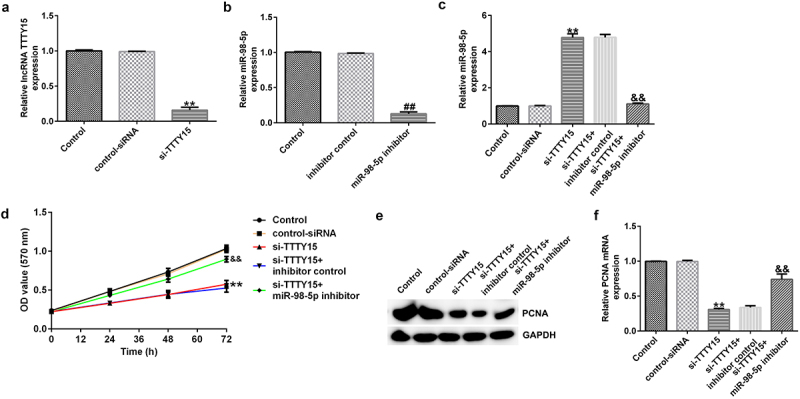
lncRNA TTTY5 suppressed miR-98-5p expression in AGS cells to regulate AGS cell proliferation.

The effects of lncRNA TTTY15 and its target miR-98-5p on AGS cell proliferation were investigated. As shown in Figure 3a, after transfection with si-TTTY15, lncRNA TTTY15 expression was clearly suppressed in AGS cell compared to that in control siRNA group, suggesting that the interference was successful. Similarly, miR-98-5p inhibitor transfection also down-regulated the level of miR-98-5p in AGS cells (Figure 3b). Loss- and gain-of-function experiments (Figure 3c) then showed that si-TTTY15 treatment could enhance miR-98-5p expression in AGS cells compared to that in control siRNA group. However, this enhancement could be partially offset by co-transfection with miR-98-5p inhibitor. Comprehensively, the above results suggest that TTTY15 reduces miR-98-5p expression in gastric cancer cells.

(A) si-TTTY15 greatly knocked down lncRNA TTTY5 expression in AGS cells. (B) Transfection efficacy of miR-98-5p after miR-98-5p inhibitor interference treatment in AGS cells. (C) miR-98-5p level in AGS cells after transfection with si-TTTY15 and/or miR-98-5p inhibitor. (D) lncRNA TTTY5 silencing greatly reduced AGS cell proliferation. lncRNA TTTY5 silencing decreased the expression of PCNA protein (E) and the expression of PCNA mRNA (F) in AGS cells.

Furthermore, the MTT assay illustrated that lncRNA TTTY15 silencing greatly reduced AGS cell proliferation compared with that in negative control siRNA (Figure 3d). Additionally, the expression of proliferation-related protein PCNA was also decreased in AGS cells after si-TTTY15 transfection (Figure 3(e,F)). Conversely, the inhibitory effects of si-TTTY15 on AGS cell proliferation could be offset by the miR-98-5p inhibitor (Figure 3(d-F)). Collectively, these results indicate that lncRNA TTTY15 knockdown reduced AGS cell proliferation by targeting miR-98-5p.

### lncRNA TTTY15 knockdown triggered AGS cell apoptosis by sponging miR-98-5p

**Figure 4.**
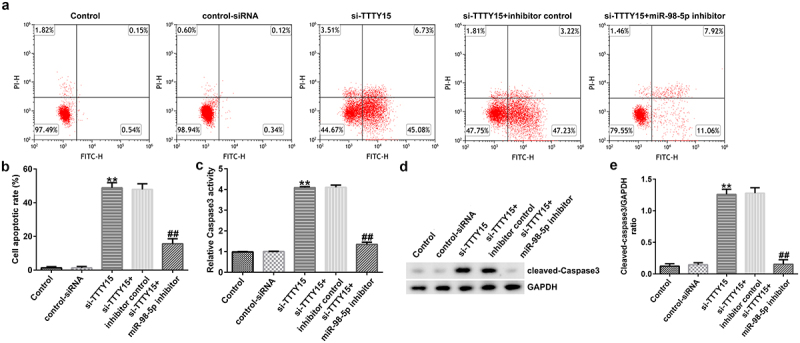
lncRNA TTTY5 induced AGS cell apoptosis by sponging miR-98-5p.

The impact of lncRNA TTTY15/miR-98-5p axis on the regulation of AGS cell apoptosis was also investigated. As shown in Figure 4(a-C), lncRNA TTTY15 knockdown significantly promoted AGS cell apoptosis and enhanced caspase-3 activity. Additionally, the pro-apoptosis protein cleaved-caspase-3 was also increased after knockdown of TTTY15 expression in AGS cells (Figure 4(d,E)). Conversely, the miR-98-5p inhibitor could reverse the promotive effects of lncRNA TTTY15 down-regulation on AGS cell apoptosis (Figure 4(a-E)). Collectively, these results indicate that lncRNA TTTY15 down-regulation promotes AGS cell apoptosis by inhibition of miR-98-5p expression.

(A) Flow cytometry analysis of the effect of lncRNA TTTY5 and miR-98-5p on AGS cell apoptosis (B) Quantified results of the flow cytometry analysis. (C) lncRNA TTTY5 silencing promoted caspase-3 activity in AGS cells by sponging miR-98-5p. Cleaved-caspase-3 protein (D) and the cleaved caspase-3/GAPDH ratio (E) in AGS cells transiently transfected with si-TTTY-5 and miR-98-5p inhibitor.

### CCND2 was negatively modulated by miR-98-5p in gastric cancer

**Figure 5.**
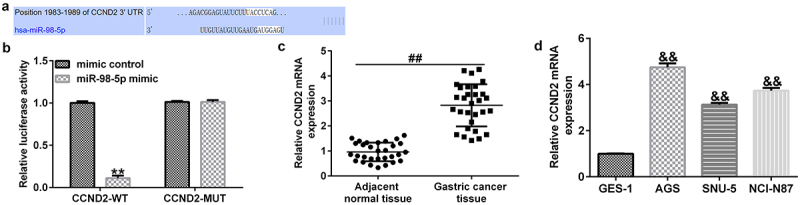
Identification of CCND2 as a downstream target of miR-98-5p in gastric cancer.

As predicted by TargetScan, CCND2 contains potential binding sites for miR-98-5p (Figure 5a). The results of the dual-luciferase reporter assay further confirmed that the miR-98-5p mimic could decrease luciferase activity in the CCND2-WT-transfected groups, while there was no significant change in the CCND2-MUT transfected groups (Figure 5b).

(A) The TargetScan bioinformatics tool predicted the binding sequences between miR-98-5p and CCND2. (B) Dual-luciferase reporter assay confirmed the target relationship between miR-98-5p and CCND2. (C) qRT-PCR analysis was used to determine CCND2 expression in tumor and non-tumor tissues obtained from 30 gastric cancer patients. (D) Ectopic expressions of CCND2 in the GES-1 cell line and gastric cancer cell lines.

Moreover, the qRT-PCR analysis revealed that CCND2 expression was clearly up-regulated in gastric cancer tumor tissues compared to that in the paracancerous tissues (Figure 5c). Similarly, CCND2 level was enhanced in gastric cancer cell lines (AGS, SNU-5, and NCI-N87) compared with that in the GES-1 normal gastric epithelial cells. This implies that CCND2 may be an oncogene involved in gastric cancer (Figure 5d).

### miR-98-5p negatively regulated CCND2 in AGS cells

**Figure 6.**
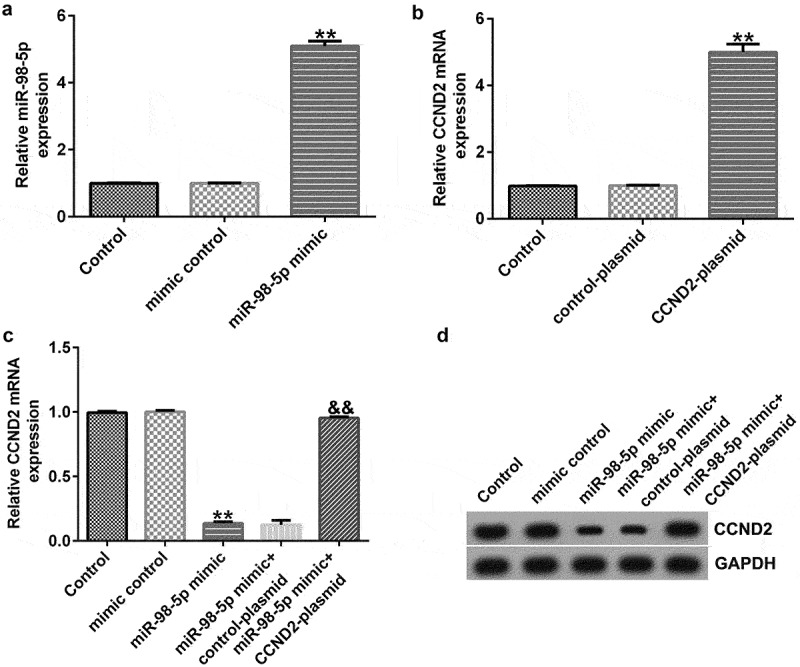
miR-98-5p negatively regulated CCND2 expression in AGS cells.

In order to confirm the regulatory relationship between CCND2 and miR-98-5p, we transiently transfected AGS cells with miR-98-5p mimic and/or CCNDS-plasmid. The results shown in Figure 6a indicate that miR-98-5p was increased in the miR-98-5p mimic group compared to that in the mimic control group. Meanwhile, CCND2 expression was up-regulated in AGS cells transfected with CCND2-plasmid compared to that in control plasmid group (Figure 6b). Finally, the co-transfection experiments revealed that the miR-98-5p mimic could reduce the level of CCND2 mRNA and protein in AGS cells and that the inhibition of CCND2 expression induced by the miR-98-5p mimic was partially offset by CCND2-plasmid transfection (Figure 6(c,D)). Comprehensively, the above results illustrate that CCND2 was negatively regulated by miR-98-5p in AGS cells, thus suggesting that CCND2 functions as a downstream target for miR-98-5p.

(A) Transfection efficacy of miR-98-5p in AGS cells. (B) Overexpression of the CCND2-plasmid construct in AGS cells. The impact of miR-98-5p transfection on the expression of CCND2 protein (C) and mRNA (D) in AGS cells.

### Up-regulation of miR-98-5p induced AGS cell apoptosis and reduced proliferation by targeting CCND2

**Figure 7.**
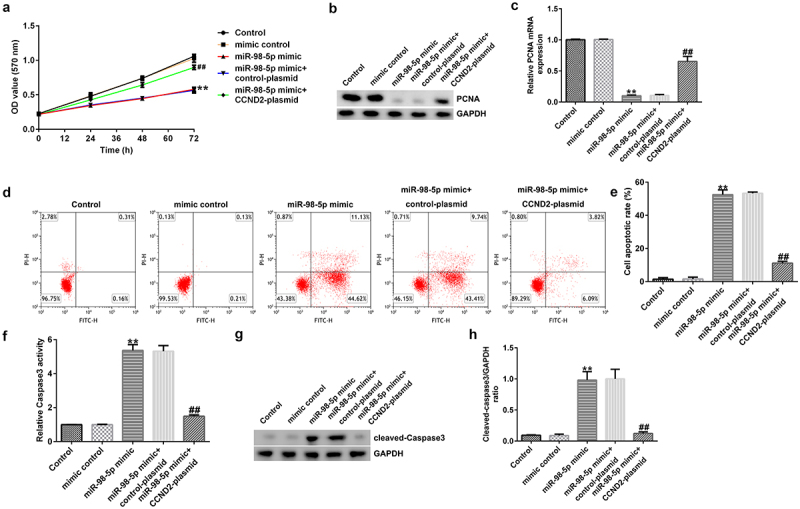
miR-98-5p overexpression suppressed AGS cell proliferation and promoted apoptosis by targeting CCND2.

To explore the effects of miR-98-5p on AGS cells, AGS cells were transfected with mimic control, miR-98-5p mimic, miR-98-5p + control plasmid, or miR-98-5p mimic + CCND2-plasmid and cultured for 48 h. Then, a series of functional experiments were conducted to verify that the miR-98-5p/CCND2 axis is involved in the regulation of gastric cancer progression. As shown in Figure 7(a-C), up-regulation of miR-98-5p inhibited AGS cell proliferation and reduced the level of the pro-proliferation protein PCNA in AGS cells. Moreover, the results in Figure 7(d-H) show that overexpression of miR-98-5p resulted in AGS cell apoptosis as well as an increase in caspase-3 activity and the level of the pro-apoptosis protein cleaved caspase-3. In contrast, the enhancing effect of the miR-98-5p mimic on AGS cell apoptosis was partially reversed by co-transfection with CCND2-plasmid (Figure 7(a-H)). Altogether, the above-mentioned results imply that miR-98-5p participates in the regulation of gastric cancer development by targeting CCND2.

(A) AGS cell proliferation was assayed by MTT assay. (B) Western blotting was used to ascertain the expression of PCNA protein. (C) The PCNA mRNA level was confirmed by PCR analysis. (D-E) Flow cytometry analysis of the AGS cell apoptosis rate. (F) Caspase-3 activity in different groups. The level of cleaved caspase-3 protein (G) and the cleaved-caspase-3/GAPDH ratio (H) were evaluated by Western blot assay.

## Discussion

The lncRNA TTTY15 coding sequence is located on the Y chromosome (chrY:12,537,650 ~ 12,860,839) and comprises 323,190 bases. As a newly discovered lncRNA, TTTY15 has been shown to play a crucial role in cardiovascular diseases. For instance, Zheng et al. [34], Chen et al. [35], Huang et al. [36], Hao et al. [37], and Zhang et al. [38] have reported that TTTY15 silencing can sponge miR-186-5p, miR-374a-5p, miR-455-5p, miR-766-5p, and let-7i-5p to mitigate hypoxia-induced vascular endothelial cell injury. Moreover, dysregulation of TTTY15 has been observed in various cancers. In colorectal cancer, TTTY15 was clearly increased in cancerous tissues and cell lines, promoting colorectal cancer cell growth and metastasis by sponging miR-29-3p [12]. In the study by Xiao et al. [13], the TTTY15 coding sequence was found to be located on the Y chromosome and its expression was up-regulated in prostate cancer tissues, thus functioning as an oncogene. Wang et al. [11] showed that TTTY15 levels were up-regulated in esophageal squamous cell carcinoma (ESCC) samples and cells, indicating that it may function as an oncogene in ESCC. On the other hand, another study revealed a tumor-suppressive role of TTTY15 in non-small cell lung cancer [14]. In the present study, we investigated the role of TTTY15 in gastric cancer. First, we found that there was pronounced up-regulation of TTTY15 in gastric cancer tissues and cell lines. However, only 30 pairs of clinical samples were included in this study, and a larger sample size may make our results more convincing, which is a research limitation of this study. Then, using siRNA technology, we knocked down TTTY15 by transfection of si-TTTY15 into gastric cancer cells. Functional experiments showed that this silencing of TTTY15 inhibited gastric cancer cell proliferation and promoted apoptosis. In this study, only flow cytometry was used to analyze cell apoptosis, and the absence of detection of apoptosis using the TUNEL assay is another limitation of the study. Collectively, for the first time, we revealed an oncogenic role of TTTY15 in gastric cancer.

The miR-98-5p coding sequence has been reported to be located on chrX:53,556,223–53,556,341 and comprises 119 bases. Recently, miR-98-5p has been found to be involved in various aspects of tumor formation and development. For instance, miR-98-5p has been shown to act as a tumor suppressor in non-small cell lung cancer [39], gastric cancer [40], glioma cancer [41], oral squamous cell carcinoma [42], ovarian cancer [43], and papillary thyroid carcinoma [44]. Xu et al. [45] observed down-regulation of miR-98-5p in gastric cancer. Meanwhile, another study showed that miR-98-5p could reduce gastric cancer cell stemness and paclitaxel chemosensitivity by targeting BCAT1 [40]. Consistent with previous studies, our study first verified the binding relationship between TTTY15 and miR-98-5p by a dual-luciferase reporter assay and RNA pull-down analysis. Furthermore, rescue experiments demonstrated that the inhibitory effects of TTTY15 silencing on gastric cancer growth could be reversed by miR-98-5p inhibitor. Additionally, overexpression of miR-98-5p by transfection with miR-98-5p mimic reduced gastric cancer cell proliferation and promoted apoptosis, implying that TTTY15 could sponge miR-98-5p to participate in the regulation of gastric cancer development.

CCND2 was predicted by the TargetScan bioinformatics tool to have potential binding sites for miR-98-5p, and it was validated as a downstream target of miR-98-5p using a dual-luciferase assay. As a component of the cell cycle machinery, CCND2 (like Cyclin D1, Cyclin D3, and Cyclin E) is a cell cycle regulatory protein [46]. Recent studies have reported that aberrant expression of CCND2 can lead to abnormal cell proliferation [47–49]. Previous studies have shown that CCND2 is aberrantly expressed in a variety of tumor tissues, such as lung cancer [50], breast cancer [51], and gastric cancer [52]. Consistent with previous research, our study found that miR-98-5p could promote gastric cancer cell apoptosis in addition to reducing cell proliferation, which could be partly offset by overexpression of CCND2. Thus, our research revealed that miR-98-5p can suppress gastric cancer development by targeting CCND2.

## Conclusion

This study showed that knockdown of lncRNA TTTY15 can down-regulate CCND2 expression by sponging miR-98-5p, thereby reducing gastric cancer progression. Thus, the lncRNA TTTY15/miR-98-5p/CCND2 axis is a potential therapeutic target for gastric cancer, and our results provide new insights for clinical treatment approaches.

## Data Availability

The datasets used and/or analyzed during the current study are available from the corresponding author on reasonable request.

## Research highlights

- lncRNA TTTY15 sponged miR-98-5p in AGS cells.
- lncRNA TTTY15 knockdown inhibited proliferation and triggered AGS cell apoptosis by sponging miR-98-5p.
- Up-regulation of miR-98-5p induced AGS cell apoptosis and reduced proliferation by targeting CCND2.
